# Athlete's Perceptions of a “Quarantine” Training Camp During the COVID-19 Lockdown

**DOI:** 10.3389/fspor.2020.622858

**Published:** 2021-01-14

**Authors:** Jad Adrian Washif, Siti Fuzyma Ayu Mohd Kassim, Philip Chun Foong Lew, Christabelle Sheau Miin Chong, Carl James

**Affiliations:** Sports Performance Division, National Sports Institute of Malaysia, Kuala Lumpur, Malaysia

**Keywords:** coronavirus, home confinement, Olympic Games, Paralympic Games, self-isolation

## Abstract

Globally, COVID-19-related movement restrictions have caused significant disruption to athlete's training and sporting competitions. “Quarantine” camps are one approach to maintain sport-specific training, whilst minimizing the risk of COVID-19 transmission between athletes and society. This cross-sectional study investigated the effects of a “quarantine” training camp on athlete's routines and wellbeing, performance support, perceived stress and sleep behaviors. A survey was completed at the end of a 30-day “quarantine” camp, by 76 elite athletes (17–46 years), predominantly (~80%) Olympic/Paralympic and/or world championship representatives. Athletes described their experiences in comparison to; pre-lockdown training and/or training during “lockdown” (immediately prior to the “quarantine” camp). Compared to “lockdown,” the “quarantine” camp revealed improvements (*p* < 0.05; 0.33 ≤ *d* ≤ 0.90) in access to sport-specific training (28.6%), recovery facilities (22.2%), nutritional choices (17.5%), mental (12.4%) and emotional (11.4%) health, training motivation (20.0%); and perceived stress (7.4%, *d* = −0.27, *p* = 0.026). The camp resulted in a lower sleep duration (−8.5%, *d* = −0.73, *p* = 0.014), but an improved global sleep behavior score (−5.6%, *d* = −0.22, *p* = 0.001). During the camp, the performance support athletes received was not different to pre-lockdown (*p* > 0.05), but there was greater sports massage (20.4%, *d* = 0.39) and physiotherapy usage (18.1%, *d* = 0.36) (both *p* < 0.05). The adverse effects of lockdown were restored during the camp. A “quarantine” camp may offer comparable training experiences to pre-lockdown training, without inducing additional perceived stress. Coaches and sporting organizations may consider this approach as part of a virus mitigation strategy, whilst maintaining sport-specific training.

## Introduction

In light of the pandemic of coronavirus disease 2019 (COVID-19), the world's sporting calendar has experienced considerable disruption. A notable example is the postponement of the Tokyo Olympics 2020, with further postponements or cancellations affecting the World Athletics Indoor Championships, UEFA European Football Championship and Badminton Thomas Cup. Travel restrictions and closures of sporting facilities have halted the regular training practices of athletes. Many athletes have been unable to perform sport-specific training, due to movement restrictions (Bok et al., 2020). Consequently, athletes have resorted to training at home, often without supervision or specialist equipment (Mon-López et al., 2020). Despite this emerging evidence demonstrating beneficial effects of home-training, the overall effectiveness of home-training for maintaining optimal levels of sport-specific conditioning remains unclear.

One option for sports seeking to continue sport-specific training, whilst controlling the spread of COVID-19, may be a “quarantine” camp. Here, athletes and support staff can be isolated from the general population for a period of time. In Malaysia, national elite athletes from several Olympic sports under the program of “*Road to Tokyo 2020*” were permitted to resume training during a 30-day quarantine-style camp in June 2020, whilst national movement restrictions (“lockdown”) remained in place. This “quarantine” camp allowed coaches and athletes to return to sport-specific training, use regular training facilities and receive performance support from sports science/medical staff within the camp. It also allows athletes to focus on the quality of training, without having to worry about training facilities and nutritious food, less travel time to the training venue and external “distractions” (e.g., media), all of which will provide a conducive training environment. However, a “quarantine” camp necessitates stringent working and living procedures to maintain a minimized risk of virus transmission. Notably, athletes and staff must be tested upon entry and may not leave the camp throughout the duration of the camp. Therefore, despite the apparent benefit of resuming sport-specific training, a “quarantine” camp may also elicit a psychological strain on athletes, who are restricted in their movements and away from family/friends (Jukic et al., 2020). Living in an isolated environment may also accentuate negative consequences of home confinement such as altered sleep patterns and poor nutrition (Pillay et al., 2020). It remains unclear therefore, whether a “quarantine” camp may be a desirable training solution in the lead up to the postponed 2020 Tokyo Olympics.

At the current time, there is no evidence detailing elite athlete's perceptions of a “quarantine” camp. Such information is warranted in order to facilitate informed decision making by coaches, sport scientists and sporting governing bodies. The present study investigated Olympic and Paralympic athlete's routines and wellbeing, performance support, perceived stress and sleep behaviors during a “quarantine” training camp, relative to the prior period of home confinement (i.e., “lockdown”) and “normal” training conditions (i.e., “pre-lockdown”). We hypothesized that a “quarantine” camp would improve athlete's perceptions of training routines and wellbeing, access to performance support, stress and sleep, compared to the prior “lockdown” and “lockdown” period.

## Methods

### Design

A cross-sectional design was adopted, using questionnaires to assess athlete's perceptions of “before” lockdown, “during” lockdown, and “during” the “quarantine” camp. All data were recorded within a 5-day period following the completion of the 30-day camp and collected using a custom-made Google Form. All athletes attended the camp for at least 23 of the 30-day duration. Data collection occurred across three training venues within the country, all of which were subjected to the same operating procedures/restrictions and requiring COVID-19 testing upon entry and exit.

### Participants

The survey was completed by 76 elite athletes (53 males and 23 females; 26 ± 5 years, range 17–46 years). All participants had between 8 and 22 years of competitive experience in their sport (15.5 ± 6.5 years). Of these athletes, ~80% have already qualified for the 2020 Olympics or have previously competed at the Olympics or World championships (i.e., highest levels of competition for each sport). Participants were from six able-bodied sports; archery (*n* = 5), badminton (*n* = 16), diving (*n* = 10), gymnastics (*n* = 4), sailing (*n* = 9), swimming (3) and nine Paralympic sports; para archery (*n* = 4), para athletics (*n* = 3), para badminton (*n* = 6), boccia (*n* = 2), para cycling (*n* = 7), para powerlifting (*n* = 2), para swimming (*n* = 1), para table tennis (*n* = 2), and wheelchair tennis (*n* = 2). The total number of participants from able-bodied sports was 47 and 29 athletes were from Paralympic sports. Participants with intellectual disabilities were not recruited. The study was conducted according to the Declaration of Helsinki. Informed consent was obtained from all athletes, with data was processed anonymously. Ethical approval was not sought as these questionnaires were comparable to those they would routinely provide as part of their official duties as national athletes (Winter and Maughan, 2009).

### Survey Questionnaire

The survey contained 6 sections; (i) athlete background and camp ratings, (ii) training routines and wellbeing, (iii) access to sport science support, (iv) perceived stress, (v) sleep behaviors, and (vi) lifestyle. Approximately 15–20 min was required to complete the full survey. To improve the response rate, the completion of parts 5 and 6 were optional, but 1–4 was compulsory. The original survey in English was translated into Malay to facilitate data collection among non-English speaking athletes. A three-step procedure was used to obtain the translation: (a) translation via *Google Translate*; (b) independent proofreading by two bilingual translators; (c) discussion and confirmation of the translation's accuracy by these two bilingual translators. A parallel comparison with the original English questionnaires was included for each question.

Section 1 pertained to athlete demographics, such as age, sex, sport, and competitive experience. Section 2 focussed on athlete's training routines and wellbeing investigating changes in training from before and during lockdown, as well as the “quarantine” camp usually emphasized in athletes. Section 2 contained nine questions relating to training facilities access, recovery facilitates access, nutritional intake, motivation, mental wellbeing, and sleep patterns. All questions were phrased: “I have been impacted, *negatively*, in the following aspects.” Athletes were asked to respond to the statement on a 5-point Likert scale, with 1 and 5 representing *strongly disagree* and *strongly agree* ratings, respectively, 1-point increments. The scores of each question in Section 2 were subsequently reversed to obtain “positive scores,” to aid interpretation and reporting of these data (e.g., a score of 5 was transformed to 1, and 4 to 2). In Section 3, the utilization of performance support (i.e., athlete interaction with sports science/medical staff during pre-lockdown training, and the “quarantine” camp) was assessed using 10 custom-designed questions. The questions are related to training monitoring, recovery practices, mental wellbeing techniques and nutrition monitoring. Athletes indicated how frequently, on a monthly basis, they utilized performance support on a 5-point Likert scale of “0 or never,” “1–2 times,” “3–4 times,” “5–6 times,” and “more than 6 times.” Section 4 utilized the Perceived Stress Scale (PSS) (Cohen et al., 1983). This involves 10 questions investigating an individual's perception of stress. It was scored on a 5-point Likert scale ranging from 0 (never) to 4 (very often). Positive scores were reversed to allow the calculation of a cumulative total score. PSS scores can range from 0 to 40, with higher scores reflecting higher levels of stress. Section 5 investigated athlete's sleep routines using the Athlete Sleep Behavior Questionnaire (ASBQ) (Driller et al., 2018). This 18-item questionnaire requires participants to indicate how often they engaged in a specific behavior on a 5-point Likert scale from 1 (never) to 5 (always). A higher score in ASBQ is indicative of poor sleep behaviors. Athletes only responded to Section 5 in relation to lockdown and the camp, as pre-lockdown was considered too far in the past for accurate recall. Section 6 investigated athlete's lifestyle habits including weekly activities and scored on a 5-point Likert scale ranging from 1 (never) to 5 (very often).

### Statistical Analysis

Raw data were downloaded from *Google Forms* and extracted into a Microsoft Excel spreadsheet (Microsoft Corporation, Redmond, WA, USA), for duplication checking and identification of missing data. Normal distribution of data were determined using the Shapiro-Wilks test. This revealed the data not to be normally distributed, therefore non-parametric analysis was adopted. Friedman's ANOVA was used to identify differences across the three time-points; pre-lockdown, lockdown and “quarantine” camp, with Wilcoxon signed rank test used as a post-hoc to identify where differences occurred. For parts of the survey where only two time-points of data were collected (e.g., ASBQ), Wilcoxon signed rank test was again adopted to identify within-subject differences. Cohen's *d* effect sizes were calculated, with the following interpretation boundaries; <0.2 (trivial), 0.2 (small), 0.5 (moderate), and 0.8 (large) with the relevant citation (Cohen, 1988). Data are presented as Mean ± SD. Responses from sections 2, 3, 4, 5, and 6 were converted to percent changes to aid interpretation and comparison. The Spearman correlation coefficient and Chi square test were used to identify the relationships between continuous (i.e., age and competitive experience) and categorical (i.e., sex) variables, respectively. Statistical analysis was performed using SPSS Statistics for Windows, version 16.0 (SPSS Inc., Chicago IL, USA), with the significance level set at *p* < 0.05.

## Results

The data of all athletes for both the compulsory and optional sections were included in statistical analysis.

Responses from Section 1 were not statistically different across the three “quarantine” camp locations, allowing a pooled analysis of data. This included no difference in athletes' ratings of access to training facilities and sports science/medical support across the three locations.

**Figure 1:**
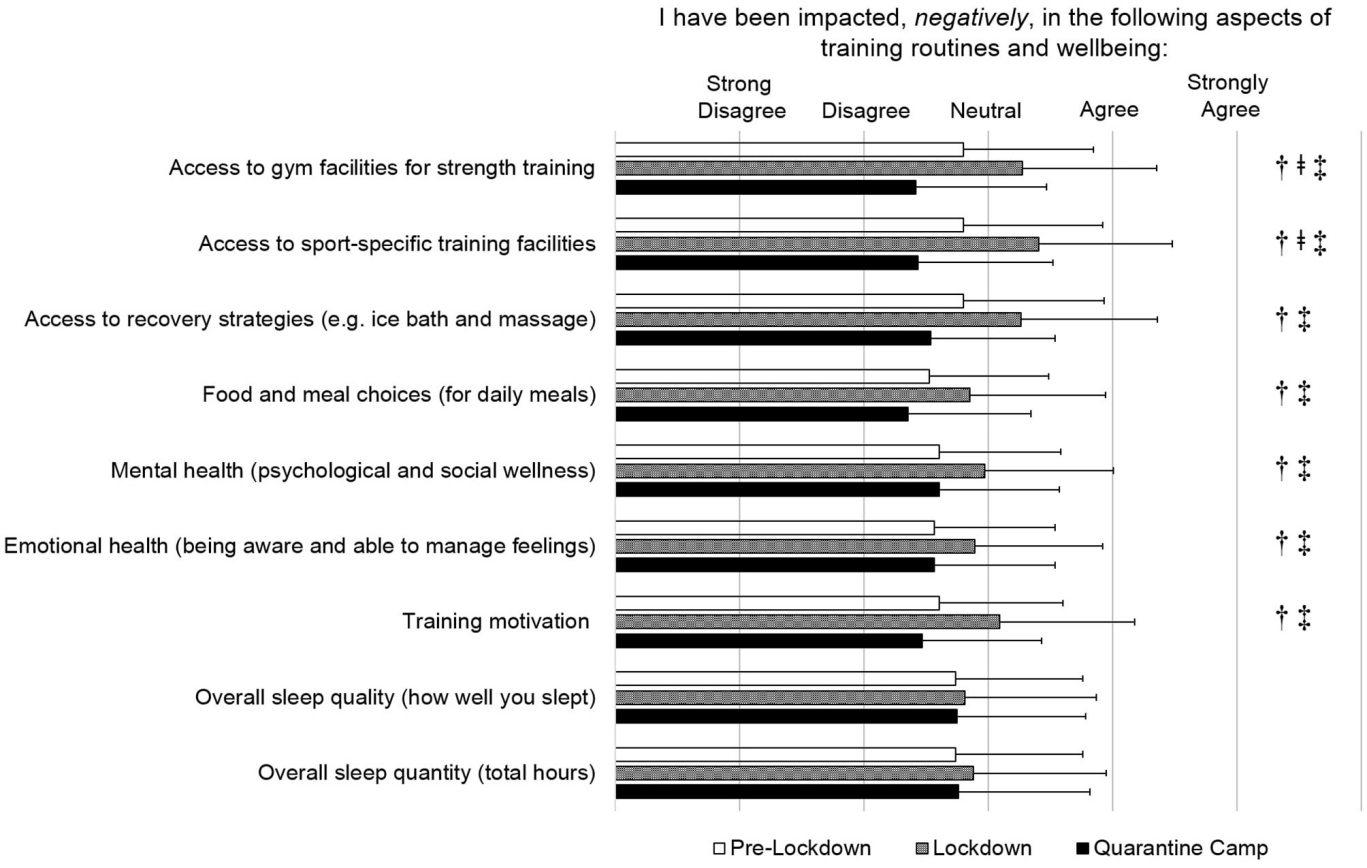
Survey section 2; athlete's training routines and wellbeing responses. All questions were phrased: “I have been impacted, negatively, in the following aspects”: The questionnaire used a 5-point scale, with 1 and 5 representing strongly disagree and strongly agree ratings, respectively, and using 1-point increments. ^†^Significant difference (*p* < 0.05) between pre-lockdown and lockdown.^
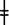
^Significant difference (*p* < 0.05) between pre-lockdown and “quarantine” camp.^‡^Significant difference (*p* < 0.05) between lockdown and “quarantine” camp.

The effects of the “quarantine” camp on training routines and athlete wellbeing (Section 2) are shown in Figure 1. A difference between pre-lockdown, lockdown and “quarantine” camp (*p* < 0.05) was observed for 7 out of 9 questions. *Post hoc* analysis found differences between lockdown (*p* < 0.05) compared to “normal” training, but these aspects were not different compared to pre-lockdown and the “quarantine” camp (*p* < 0.05). Differences between pre-lockdown and lockdown were identified for; access to gym facilities for strength training (pre-lockdown to lockdown: −16.9%, *d* = −0.45; lockdown to “quarantine” camp: 26.1%, *d* = +0.80), access to sport-specific training (pre-lockdown to lockdown: −21.6%, *d* = −0.55; lockdown to “quarantine” camp: 28.6%, *d* = +0.90), access to recovery services (−16.4%, *d* = −0.41; 22.2%, *d* = +0.69), food and meal choices (−13.0%, *d* = −0.32; 17.5%, *d* = +0.48), mental health (−14.1%, *d* = −0.37; 12.4%, *d* = +0.37), emotional health (−12.8%, *d* = −0.33; 11.4%, *d* = +0.33) and training motivation (−18.7%, *d* = −0.47; 20.0%, *d* = +0.60). Sleep quality and quantity did not change throughout the assessed periods (rated between “disagree” and “neutral” throughout).

**Figure 2:**
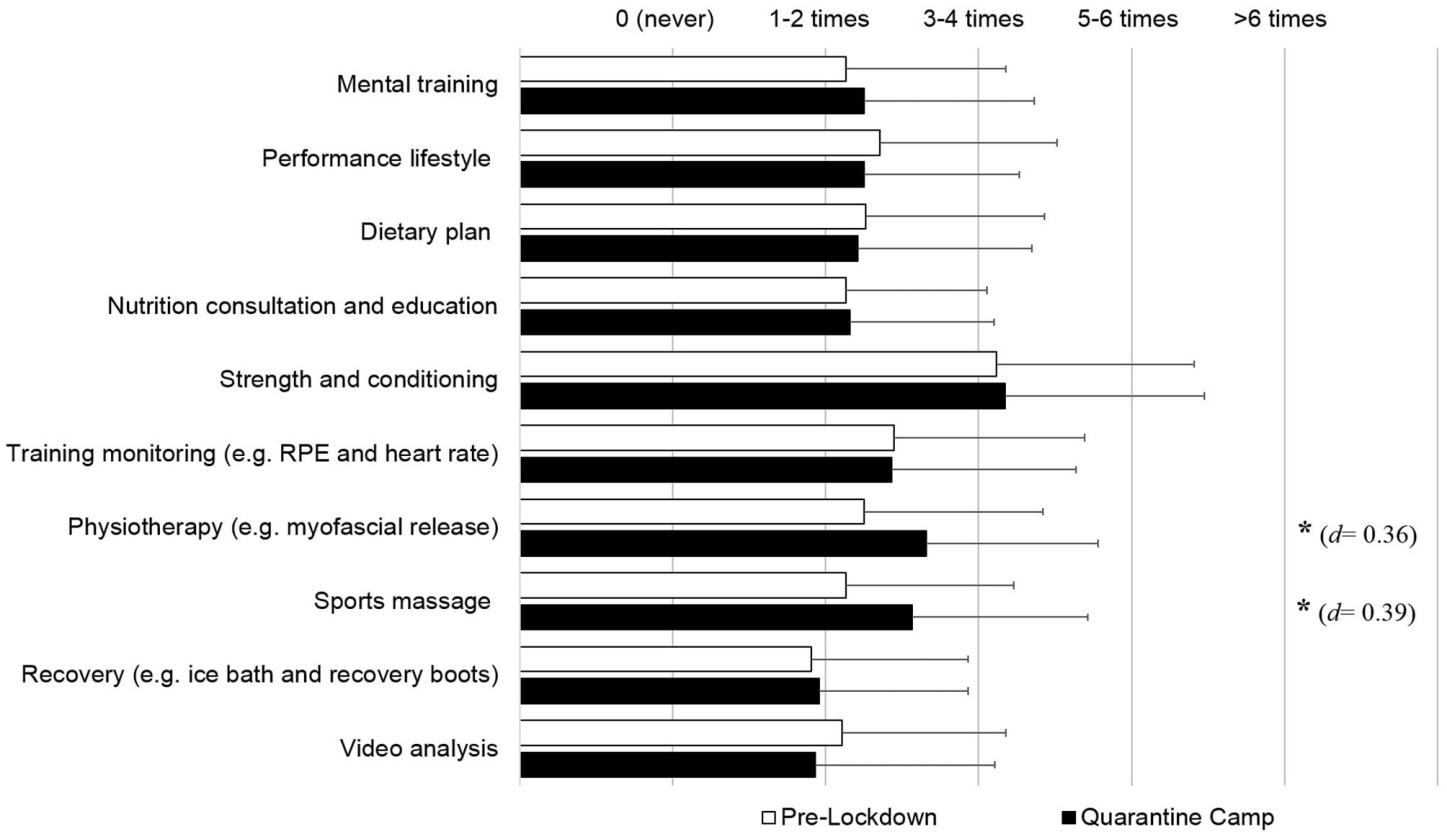
Survey section 3; Utilization of performance support pre-lockdown and during the “quarantine” camp. Athletes indicated how frequently, on a monthly basis, they utilized performance support on a 5-point scale of “0 or never,” “1–2 times,” “3–4 times,” “5–6 times,” and “more than 6 times.” * Significant difference (*p* < 0.05) between pre-lockdown and “quarantine” camp.

Figure 2 presents performance support across all timescales (Section 3). Sports massage (20.4%, *d* = 0.39) and physiotherapy (18.1%, *d* = 0.36) usage increased during the “quarantine” camp (*p* < 0.05). The remaining aspects of performance support were not statistically different between pre-lockdown and “quarantine” camp.

**Figure 3:**
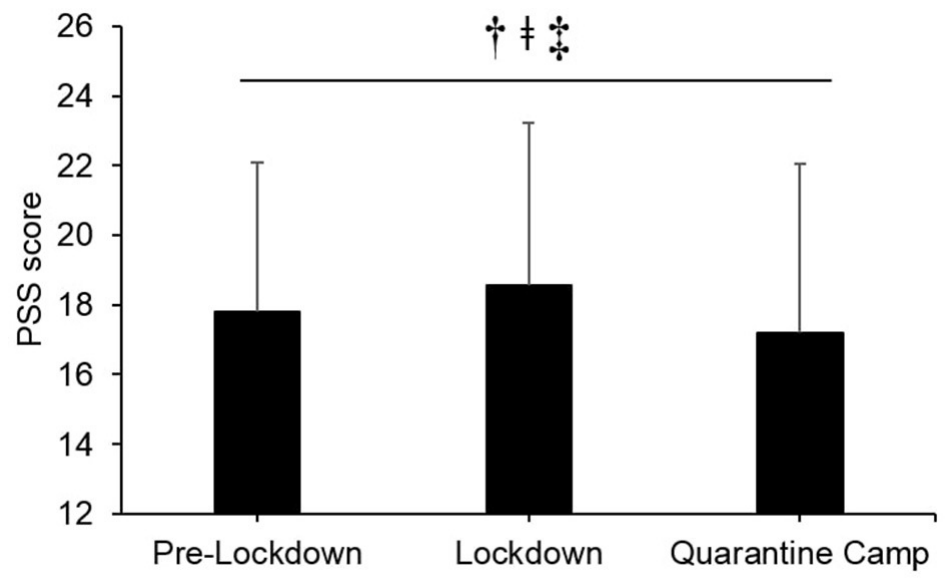
Survey section 4; Comparison in Perceived Stress Scale (PSS) among pre-lockdown, lockdown, and “quarantine” camp. Responses were scored on a 5-point scale ranging from 0 (never) to 4 (very often). Positive scores were reversed to allow the calculation of a cumulative total score. ^†^Significant difference between pre-lockdown and lockdown.^
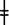
^Significant difference between pre-lockdown and “quarantine” camp.^‡^Significant difference between lockdown and “quarantine” camp.

A difference in the average PSS total score (Section 4) was observed between pre-lockdown, lockdown and “quarantine” camp (χ^2^ = 11.006, *p* = 0.004, Figure 3). *Post hoc* analysis indicated differences between pre-lockdown and lockdown (*Z* = −2.661, *p* = 0.008), between pre-lockdown and “quarantine” (*Z* = −2.224, *p* = 0.026) and between lockdown and “quarantine” (*Z* = −3.300, *p* = 0.001). The PSS scores for pre-lockdown, lockdown, and the “quarantine” camp were 17.8 ± 4.3 pt, 18.6 ± 4.6 pt, and 17.2 ± 4.9 pt, respectively. The results indicate increased stress level during lockdown (+4.4%, *d* = 0.17) which reduced during the “quarantine” camp (−7.4%, *d* = −0.27) compared to pre-lockdown. Neither age, sex, nor competitive experience were correlated with the PSS total score for pre-lockdown, lockdown, and “quarantine” camp (*p* > 0.05; *r* = −0.22 to 0.62).

Sleep duration (Section 5) was higher during lockdown compared to the “quarantine” camp (*Z* = −2.446, *p* = 0.014, −8.5%, *d* = −0.73). The mean change was from 8:08 ± 1:27 during lockdown, to 7:39 ± 0:44 h during the “quarantine” camp. There was a difference in the ABSQ global score between lockdown and the “quarantine” camp (*Z* = −4.470, *p* = 0.001, −5.6%, *d* = −0.22). A higher score during lockdown (37.0 ± 9.1) indicated impaired sleep behaviors during lockdown, relative to “quarantine” camp (34.9 ± 9.7). There were no correlations for age, sex, and competitive experience with the ABSQ scores during lockdown and “quarantine” camp (*p* > 0.05, *r* = −0.09 to 0.68).

**Figure 4:**
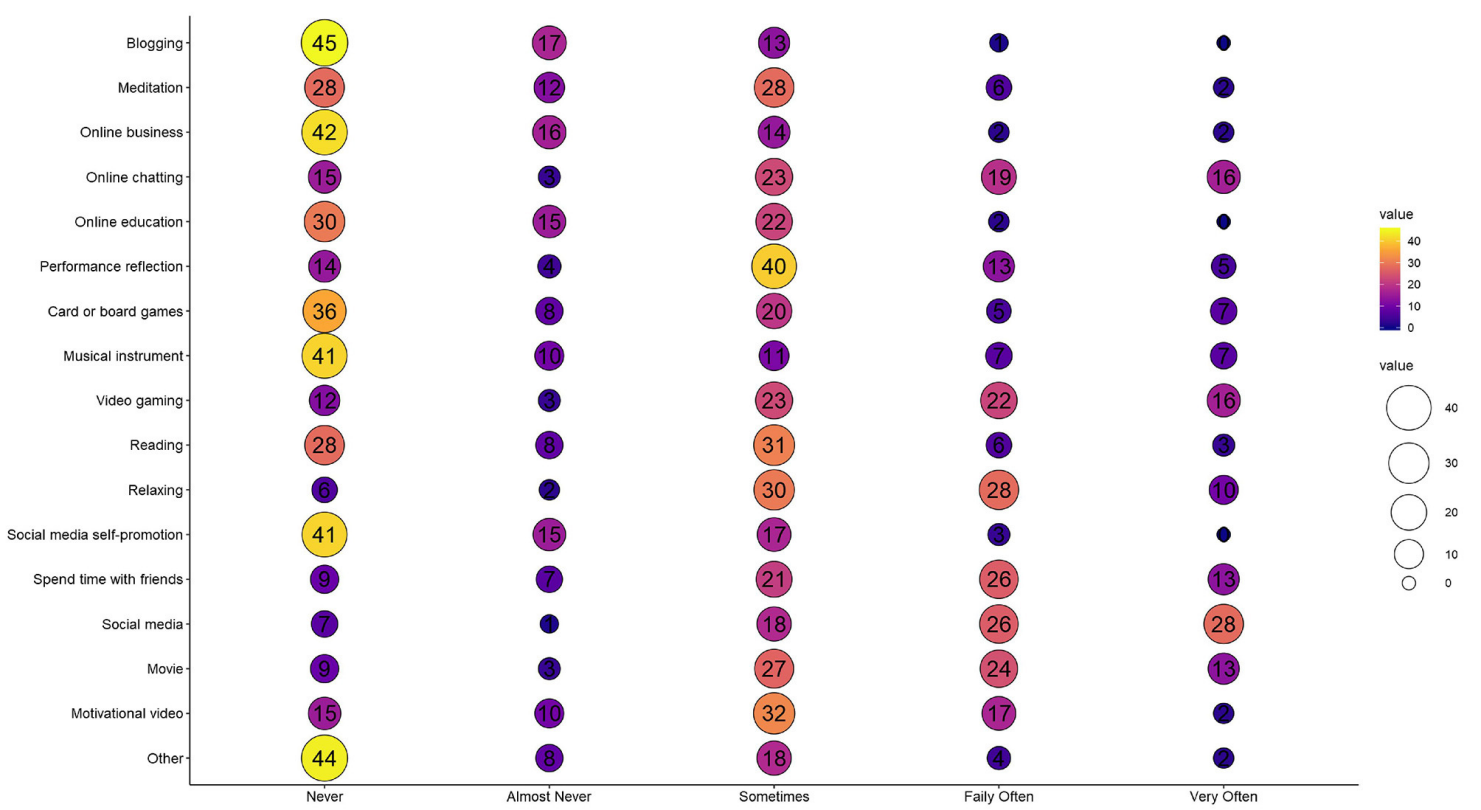
Survey section 6; total responses to athlete's lifestyle habits during the “quarantine” camp. Responses were scored on a 5-point scale ranging from 1 (never) to 5 (very often).

Figure 4 shows the weekly activities of athletes (Section 6) during the “quarantine” camp. An effect for sex was found in “watch movie” (χ^2^ = 25.183, *p* = 0.001), “use social media” (χ^2^ = 14.371, *p* = 0.002), and “online education” (χ^2^ = 10.917, *p* = 0.028). However, all other aspects of weekly activities were not statistically different.

## Discussion

The current study is the first to report athlete perceptions during a “quarantine” training camp. During lockdown, we observed negative effects on athlete's access to training and recovery facilities, increased mental and emotional stress, fewer nutritional choices, reduced training motivation and increased perceived stress (all *small* to *moderate* effects). However, our findings indicate that these detrimental effects were reversed during the “quarantine” camp (all *small* to *large* effects). The “quarantine” camps allowed athletes to complete sport-specific training with other athletes, supported by coaches and performance support staff. Compared with lockdown, athlete's training routines, performance support, and perceived stress improved during the camp, along with sleep behaviors. These outcomes confirmed our hypotheses that the negative effects associated with COVID-19 pandemic lockdown would be reversed during a “quarantine” training camp.

We observed negative effects of lockdown on athletes' mental (−14.1%) and emotional health (−12.8%), and training motivation (−18.7%) (all *small* effects). This finding concurs with Pillay et al. (2020) who reported one in two athletes to experience depression, feelings of energy loss and a lack of training motivation during lockdown. More recently, Ammar et al. (2020a) have also reported an increased number of people to have experienced negative effects on mental wellbeing and emotional status (10–16.5%, *small* to *moderate* change) as a result of home confinement. Such negative states may be induced by nervousness, preoccupation and apprehension caused by an individual's perception of the situation (Trigueros et al., 2019). This is likely related to the current COVID-19 pandemic, as athletes experience uncertainty about their return to competition and their performance level (Andreato et al., 2020). We also observed reduced meal or food choices during lockdown, compared with pre-lockdown (Figure 1). Recent literature has reported that the confinement associated with the COVID-19 response (e.g., self-isolation or lockdown) can lead to poor diet management (Ammar et al., 2020c; Pillay et al., 2020). Many athletes may perceive eating a wide and varied diet to be an important aspect of their daily routines, which can help to maintain immunity (Yousfi et al., 2020). Therefore, a perceived limitation of nutritional choices is a likely stressor. However, the camp environment improved nutritional choices for athletes (17.5%), whilst athletes also undertook more nutrition and dietary consultations with performance staff (Figure 2). Overall, mental wellbeing, emotional wellbeing, training motivation, and nutritional choices were improved during the “quarantine” camp as athletes had full access to all facilities and “regained” their “normal” daily routines. It highlights why a “quarantine” camp during such a period of “catastrophe” is valuable to facilitate “normal life” of athletes, while allowing a systematic performance support to be implemented to enhance athletes' training routines and psychological well-being.

Sleep quality and quantity appeared unaffected during lockdown, compared to pre-lockdown. Sleep quantity during lockdown (>8 h) was higher than during “quarantine” camp (<8 h), although both remain within the recommended duration for athletes (i.e., 7–9 h) (Watson, 2017). Similarly, increased sleep quantity during lockdown (from 7.2 to 8.0 h) was also observed among handball players, ascribed by mobility restrictions (Mon-López et al., 2020). Nevertheless, the sleep quantity during the “quarantine” camp appears consistent with current literature reporting elite athletes achieving <8 h of sleep per night (Lastella et al., 2015). However, recent data has highlighted the relationship between perceived stress and sleep quality (Altena et al., 2020), which is supported by other recent studies conducted in both China (Li et al., 2020) and Italy (Casagrande et al., 2020) during the COVID-19 lockdown. Whilst we did not observe reduced sleep quality during lockdown, the ASBQ global score indicated improved sleep behaviors during the “quarantine” camp compared to lockdown (−5.6%, *small* effect). This indicates that athlete's sleeping habits were not impacted on by the camp environment, whilst athletes could not sleep in their own bed.

Our data revealed increased perceived stress level during the lockdown, compared to pre-lockdown (4.4%, *trivial* effect), (Figure 3). Our data are in agreement with recent research among general population (Ammar et al., 2020b) and Italian athletes (di Fronso et al., 2020). The pandemic has highlighted multiple potential stressors and individual circumstances likely determine the contributing role of each to athlete's perceived stress. COVID-19 has created new strains on elite athletes, who were associated with greater symptoms and disorders of mental health than the general population (Reardon et al., 2020). Event cancellation, contract revision with clubs, among others were likely to contribute to severe psychological state in athletes (di Fronso et al., 2020). However, we observed a reduced stress during the “quarantine” camp (−7.4%) when athletes were able to training “normally.” For centuries, “quarantine” has successfully been used to control the spread of contagious viruses (Hawryluck et al., 2004). Individuals placed in “quarantine” have their movement restricted from other people and report stress from the changes in the living conditions, as well as negative thoughts about one's own health or those in close proximity (Hawryluck et al., 2004). In contrast, the current study found that the “quarantine” camp to improve the perceived stress level of athletes, possibly due to a different nature of “quarantine.” In the “quarantine” camp, despite stringent operating procedures, athletes lived more “normally” and were able to perform daily routines as an athlete whilst interacting with other athletes and staff. This differs from lockdown, whereby individuals were disconnected from most individuals, including family and friends. It should also be considered that athletes may perceive the threat of transmission of the virus to be lower within the camp, as they remain physically disconnected from the wider public. In summary, a camp environment resulted in a reduction in perceived stress for athletes, compared to lockdown, which is an important observation for those planning training in the lead-up to the delayed Tokyo 2020 Olympics.

Elite athletes are routinely surrounded by a team of professionals dedicated to maximizing performance (Heidari et al., 2019). We found similar usage of performance support services in the camp, compared to pre-lockdown (Figure 2). Interestingly, athletes utilized greater masseur and physiotherapist support during the “quarantine” camp, possibly to enhance recovery from sport-specific training following the generic training that occurred during lockdown. Away from training, a high proportion of athletes spent most of their time using social media (77%), watching movies (68%), and talking with friends (67%) during the camp (Figure 4). This supports similarly high social media use and watching of television as the primary “home activities” recently reported among athletes during home confinement (Pillay et al., 2020). Those considering implementing “quarantine” camps may therefore wish to consider what other types of socially-distanced activities may be provided as options to athletes residing within a camp.

It should be noted that these data were obtained on a single occasion at the end of the “quarantine” camp. There is therefore the potential for recall bias and subjectivity. However, it was not possible to collect information during earlier periods given the unpredictable development of the pandemic. We also included “bespoke” questions within our survey (e.g., Section 2), for which we cannot demonstrate validity and reliability. Nevertheless, these questions were generic and we believe unlikely to be misinterpreted and as such, enhances our understanding of athlete's experiences within the camp.

To sum up, we observed a variety of negative effects in elite athletes that are associated with home confinement. However, these effects were not maintained during the “quarantine” camp, which appeared to improve aspects of athlete's routines and wellbeing, perceived stress, performance support and sleep behavior. The outcomes of the current study have important implications for policy makers, governing bodies, coaches, sports scientists considering implementing “quarantine” camps in the months leading up to the delayed Tokyo 2020 Olympic Games.

### Practical Implications

- A “quarantine” training camp offers one option for athletes to maintain “normal” training practices, with access to coaching, sports science and medical staff, whilst minimizing the risk of COVID-19 transmission with wider society.
- Following a period of home confinement, athletes may utilize greater physiotherapy/masseur support than usual to assist with the training and recovery process.
- Caution should be taken when interpreting these findings in the context of planning camps that exceed a duration of 30 days.

## Data Availability Statement

The raw data supporting the conclusions of this article will be made available by the corresponding author, upon reasonable request.

## Ethics Statement

The questionnaires were comparable to those the participants would routinely provide as part of their official duties as national athletes. This study was conducted in accordance with high ethical standards, and approved retrospectively by the institutional review board of Institut Sukan Negara (004/2020-005/2020). The participants provided their written informed consent to participate in the study.

## Author Contributions

JW: original planning, overall contribution, study conceptual, study planning, data collection, data collation, data analysis, data interpretation, manuscript (first draft), manuscript editing, and critical revision of manuscript. SM: original planning, content contribution, study planning, data collection, manuscript (first draft), and manuscript editing. PL and CC: content contribution, study planning, data collection, manuscript (first draft), and manuscript editing. CJ: overall contribution, study conceptual, study planning, data collection, data interpretation, manuscript (first draft), manuscript editing, and critical revision of manuscript. All authors contributed to the article and approved the submitted version.

## Conflict of Interest

The authors declare that the research was conducted in the absence of any commercial or financial relationships that could be construed as a potential conflict of interest.
